# Impact of COVID-19 pandemic on obesity among adults in Jordan

**DOI:** 10.3389/fnut.2023.1114076

**Published:** 2023-01-20

**Authors:** Taha Rababah, Muhammad Al-U'datt, Malak M. Angor, Sana Gammoh, Rana Rababah, Ghazi Magableh, Ali Almajwal, Yara AL-Rayyan, Numan AL-Rayyan

**Affiliations:** ^1^Department of Nutrition and Food Technology, Jordan University of Science and Technology, Irbid, Jordan; ^2^Nutrition and Food Processing Department, Al-Huson College, Al-Balqa Applied University, Salt, Jordan; ^3^Industrial Engineering Department–Yarmouk University, Yarmouk University, Irbid, Jordan; ^4^College of Applied Medical Sciences, King Saud University, Riyadh, Saudi Arabia; ^5^College of Agriculture and Life Sciences, University of Wisconsin-Madison, Madison, WI, United States; ^6^School of Medicine and Public Health, University of Wisconsin-Madison, Madison, WI, United States; ^7^National Agricultural Research Center, Al-Baqa'a, Jordan

**Keywords:** obesity, COVID-19, lifestyle, Jordan, BMI, pandemic, eating habits

## Abstract

COVID-19 is a severe acute respiratory syndrome that mainly affects the human respiratory system. Unhealthy nutritional habits and obesity are expected as consequences of protective measures including quarantine. Obesity, in its growing prevalence, is a worldwide health issue associated with worsening health conditions. This is a cross-sectional study to assess the impact of the COVID-19 pandemic on obesity among Jordanian adults and across epidemiological statuses. Participants were randomly selected, and the survey was distributed on social media networking sites. A total of 672 subjects were surveyed and participated in the study between March and June 2021 *via* Google Form questionnaire. The results indicated that 74.4% of participants reported that they did not do any physical activity, and 43.5% changed their lifestyle and eating habits for the worse. During the COVID-19 pandemic, almost half of the participants reported an increase in hunger, consuming 3–4 meals/day, and consuming < 1 liter of water/day. Additionally, more than half of the participants reported no change in fat, cereals, and protein consumption, 46.4 % had no change in fruit and vegetable consumption, and 50.6% increased their consumption of sweets. Our results showed a significant increase in the self-reported BMI categories during the COVID-19 pandemic for all ages (*p* < 0.001). Change in weight and BMI was significantly associated with marital status, education level, living place, family size, family working members, and working status. Participants across all epidemiological statuses displayed a statistically significant increase in BMI. This study was conducted to observe the impacts of the COVID-19 pandemic on health behaviors and obesity among Jordanian adults and across epidemiological statuses. We found that there were significant negative changes in the lifestyle (physical activity) and eating behaviors of Jordanians during the COVID-19 quarantine which in turn increased their body weight and changed the obesity rate.

## 1. Introduction

COVID-19 is a severe acute respiratory syndrome caused by one of the novel viruses, coronavirus-2 (SARS-CoV-2), which mainly affects the human respiratory system (1). In December 2019, it was reported that SARS-CoV-2 was rapidly spreading from Wuhan City of Hubei, Province of China, then to the rest of the world (2). The World Health Organization (WHO) declared COVID-19 a global pandemic on March 11, 2020, due to the significant increase of infected people and the increase of new infections (3). As a result, the countries of the world started strict national policies and decided to close their borders to contain and prevent the prevalence of the virus. Since Jordan was affected by the COVID-19 pandemic like most of the world, it took early cautious steps that significantly helped in controlling the disease. Extensive policy measures were taken, such as extensive contact tracing, quarantine of asymptomatic contacts, and hospital isolation. This was supported by a strict countrywide lockdown and a nightly curfew. The borders were closed and all travel to and from Jordan was stopped on March 15, 2020 (4, 5). Additionally, all the national institutions, including schools, universities, recreation centers, coffee shops, and grocery stores, were closed until further notice. Only hospitals and vital centers were allowed to open during the curfew. A Royal Decree was issued approving the application of the National Defense Law on March 17, 2020 (5). By April 8, 2020, the confirmed cases of COVID-19 in Jordan were 358 with 6 deaths (6).

The restricted measures of the pandemic represent a significant impact on human health causing sudden lifestyle changes through social distancing measures, stay at home orders, and economic hardship. Media reports showed dramatic shifts in life during quarantine (7). The main consequences of quarantine-induced stress are changes in lifestyle, including grocery shopping patterns which result in buying huge quantities of food items leading to more food consumption. Other changes in lifestyle included changes in sleep patterns, physical activity as well as nutritional habits. Weight gain and obesity can be due to the changes in nutritional habits caused by reduced availability of goods, limited access to food caused by restricted store opening hours, and switches to unhealthy foods (8, 9).

Particularly, the global prevalence of overweight and obesity has increased since 1980. By 2019, almost a third of the world's population was known to be overweight or obese (10). Obesity is a major risk factor for diabetes (11), cardiovascular disease (CVD) (11, 12), cancers (13), and poor mental health, all of which negatively affect the quality of life and healthcare costs (14). Before the pandemic, obesity was consistently increasing, reaching a worldwide prevalence of 11% for men and 15% for women (15). In Jordan, it was reported in research conducted in 2008 that the age-standardized prevalence rate of obesity among adults in Northern Jordan was 28.1% in men and 53.1% in women. The same study predicted an increase in the obesity rate observed in the last 10 years (16). In 2017, according to the International Diabetes Federation using waist circumference (WC) criteria, the age-standardized prevalence of obesity in Jordan was 60.4% among men and 75.6% among women (17).

Individuals with obesity are at higher risk for adverse outcomes of COVID-19 and home quarantining measures have also exacerbated factors that predispose people to obesity (18). Our research question was how did the pandemic and its associated lifestyle changes impact weight gain and healthy behaviors. The aim of this study is to assess the impact of the COVID-19 pandemic on obesity and to determine the associated factors of obesity among the adult population in Jordan.

## 2. Methods and materials

### 2.1. Study design and participants

A descriptive cross-sectional study was conducted to assess the impact of the COVID-19 pandemic on obesity among Jordanian adults (age ≥18 years). The study was conducted between March and June 2021. The questionnaire was formatted into Google Forms; an internet-based software commonly used for data collection. Participants were invited to enroll in this online survey by on pages followed or viewed by many Jordanians including the pages of all 12 Jordanian Governments for distribution on social media networking sites (Facebook and WhatsApp).

The only criteria that were used were that each participant had to be living in Jordan and be about 18 years old. Eligible individuals were informed before participating about the purpose of the study, the procedure, and the time needed to complete the questionnaire as well as the voluntary nature of participation. The informed consent was written in simple and understandable terms on the first page of the questionnaire and respondents were ensured of that all of their responses were anonymous. Before enrollment, the participants indicated their consent in participating to proceed with the questionnaire. Considering the nature of the web-based Google form surveys, the participants were instructed to fill out the questionnaire with probity after fulfilling the eligibility criteria, consenting to voluntary participation, and filling it only once. We did not provide any form of compensation to the participants upon their involvement in the study. The effectiveness and efficiency of using social media such as Facebook for the recruitment of research participants in various medical studies came into evidence through a recent systematic review of 109 published articles (19). The sample size was calculated using a web-based sample size calculator (Raosoft^®^). The sample size was 672 participants. Participants were instructed to use the period before March 2020 for the questions regarding the period before the COVID-19 quarantine and their information on the date that they completed the questionnaire during the quarantine.

### 2.2. Validity, internal consistency, and reliability

The questionnaire was based on Radwan et al. with some modifications (20). The questionnaire (originally written in English) was translated into Arabic using a forward-backward translation technique. The questionnaire was independently validated in the Radwan et al. study and utilized to collect similar data in the UAE. The questionnaire was reviewed by a group of academic experts at the Jordan University of Science and Technology (JUST), then a pre-test was performed by 15 individuals to assess the content validity and to identify any ambiguous questions to make appropriate modifications. Both UAE and Jordan communities are Arabic speakers with similar cultures, thus no language differences were found. The study was approved by the Institutional Review Board at King Abdallah University Hospital and the JUST (IRB Number, Ref 134-2021). In addition, Cronbach's alpha coefficient was applied to test the internal consistency (reliability) of the survey items. Acceptable internal consistency was shown; Cronbach's alpha was 0.712.

### 2.3. Study instrument

A self-administrated questionnaire, including 26 multiple-choice questions, was designed using a Google Form in Arabic. This survey was grouped into four main sections and contained questions on lifestyle and dietary habits before and during the COVID-19 quarantine. The first part of the questionnaire collected socio-demographic information. In the second part of the questionnaire, the anthropometric measurement (self-reported weight before and during the COVID-19 quarantine and the height of participants) was then introduced. The presence of chronic diseases and lifestyle practices during the COVID-19 quarantine (smoking status, sleep, physical activity, and sitting hours using screens such as televisions, mobiles, or computers) were introduced in the third part. Finally, changes in dietary habits during the COVID-19 quarantine were introduced in the last part of the questionnaire. A translated English version of the questionnaire was also provided to participants included in the Supplementary material for reference.

### 2.4. Statistical analysis

Data is represented as frequency and percentages (%) for categorical variables. Data is presented as mean± standard deviation (SD) for continuous variables. Statistical analysis was conducted using IBMSPSS^®^ 25.0 for Windows. The Kolmogorov-Smirnov test of normality was applied, and since the data did not support parametric assumptions, the Kruskal-Wallis test and the Mann-Whitney *U*-test were performed when applicable. For multiple group comparisons, Bonferroni correction was applied, and the results were evaluated accordingly. The level of significance was defined as *p* < 0.05. We calculated BMI using the formula (weight in kg)/ (height in meters)^2^. A paired *t*-test was also performed to analyze individual participants' change in BMI and weight.

## 3. Results

### 3.1. Participants

A total of 1025 people were recruited for the study. A total of 149 individuals were not eligible (age <18 years), 130 individuals refused to participate in the study, and 74 individuals did not complete the survey. Of the 672 participants included in this survey, the sample distribution was 1,919, 223, and 130 participants from the Northern, Middle, and Southern regions of Jordan, respectively.

### 3.2. Sociodemographic characteristics

Table 1 shows that there was a significant difference between underweight, normal weight, overweight, and obese participants during the COVID-19 quarantine between age categories (*p* = 0.000). Obesity was significantly associated with marital status with married participants being the most likely to be obese, 25.8% (*p* < 0.001). Most of the participants who have a normal weight (i.e., BMI = 18.5–24.9) were bachelor's degree holders, with 47.7% of bachelor's degree holders having normal weight (*p* = 0.001). Furthermore, obesity was significantly related to working status (*p* < 0.001). Participants who continued going to work as they did before COVID-19 had the highest prevalence of obesity (24.7%), followed by participants working from home (21.9%) and the unemployed (18.9%).

**Table 1:** Relation between socio-demographic characteristics and obesity (*n* = 672).

**Variable**	**Category**	Total^*^ N% ***N*** **= 672**	**Underweight** ***N*****%** ***N*** **= 33**	**Normal weight *N*%** ***N*** **= 293**	**Overweight** ***N*****%** ***N*** **= 220**	**Obese *N*%** ***N*** **= 126**	***p*** **-value**
**Age**							
	18–25	220 (32.7)	27 (12.2)	116 (52.7)	55 (25.0)	22 (10.0)	0.000
	25–35	301 (44.8)	6 (2.0)	143 (47.5)	91 (30.2)	61 (20.3)	
	35–60	149 (22.2)	34 (22.8)	72 (48.3)	43 (28.8)	
	≥60	2 (0.3)	0	0	2 (100.0)	0	
**Gender**							
	Male	256 (38.1)	9 (3.5)	100 (39.1)	96 (37.5)	51 (19.9)	0.080
	Female	416 (61.9)	24 (5.8)	193 (46.4)	124 (29.8)	75 (18.0)	
**Marital status**							
	Single	357 (53.1)	30 (8.4)	183 (51.2)	99 (27.7)	45 (12.6)	0.000
	Married	302 (44.9)	3 (0.9)	104 (34.4)	117 (38.7)	78 (25.8)	
	Divorced or Widow	13 (1.6)	0	6 (46.2)	4 (30.8)	3 (23.0)	
**Education**							
	Primary/secondary school	61 (9.1)	0	16 (26.2)	27 (44.3)	21 (34.4)	0.001
	Bachelor	482 (71.7)	29 (6.1)	230 (47.7)	148 (30.7)	75 (15.6)	
	Graduate studies	127 (18.9)	4 (3.1)	47 (37.0)	45 (35.4)	31 (24.4)	
**Region**							
	North region	319 (47.5)	19 (6.0)	155 (48.6)	96 (30.1)	49 (15.4)	0.034
	Middle region	223 (33.2)	12 (5.4)	90 (40.4)	73 (32.7)	48 (21.5)	
	South region	130 (19.3)	2 (1.5)	48 (36.9)	51 (39.2)	29 (22.3)	
**Residence**							
	Urban	450 (67.0)	20 (4.4)	213 (47.3)	136 (30.2)	81 (18.0)	0.046
	Rural	222 (33.0)	13 (5.9)	80 (36.0)	84 (37.8)	45 (20.3)	
**Family size**							
	< 4	128 (19.0)	7 (5.5)	58 (45.3)	44 (34.4)	19 (14.8)	0.000
	4–6	343 (51.0)	22 (6.4)	166 (48.4)	99 (28.9)	56 (16.3)	
	≤ 7	201 (29.8)	4 (2.0)	69 (34.3)	77 (38.3)	51 (25.4)	
**Income JD**							
	< 300	95 (14.1)	2 (2.1)	52 (54.8)	27 (28.4)	14 (14.7)	0.049
	300–800	358 (53.3)	21 (5.9)	162 (45.3)	109 (30.4)	66 (18.4)	
	>800	219 (32.6)	10 (4.6)	79 (36.0)	84 (38.4)	46 (21.0)	
**Working family member**							
	1–3	614 (91.4)	32 (5.2)	264 (43.0)	202 (32.9)	116 (18.9)	0.004
	4–6	51 (7.5)	1 (2.0)	23 (45.1)	17 (33.3)	10 (19.6)	
	≥7	7 (0.9)	0	6 (85.7)	1 (14.3)	0	
**Working status**							
	Student	191 (28.4)	20 (10.5)	100 (52.3)	49 (25.7)	22 (11.5)	0.000
	Unemployed	143 (21.3)	5 (3.5)	63 (44.0)	48 (33.6)	27 (18.9)	
	Working from home	105 (15.6)	3 (2.9)	39 (37.1)	40 (38.1)	23 (21.9)	
	Going to work as before COVID-19	170 (25.3)	4 (2.4)	72 (42.3)	52 (30.6)	42 (24.7)	
	Work stopped during COVID-19	52 (7.7)	1 (1.9)	18 (34.6)	27 (51.9)	6 (11.6)	
	Retired	11 (1.6)	0	1 (9.1)	4 (36.4)	6 (54.5)	

* Data are presented as frequency N (%). Statically Significant considered at p < 0.05.

Our results showed significant differences in obesity prevalence between regions (*p* < 0.05). The Southern region of Jordan had the highest prevalence of overweight (39.2%) and obesity (22.3%) Family size was significantly associated with obesity (*p* < 0.000), in which participants with large family size (family members ≥7) had a higher prevalence of obesity at 25.4%. Furthermore, the number of working family members also had a significant association with the prevalence of obesity as well (*p* = 0.004).

### 3.3. Lifestyle characteristics

Table 2 presents the lifestyle characteristics of the study population before and during the COVID-19 quarantine. There was a change in participants' sleep patterns before and during COVID-19 with a decrease in sleep hours per night. Consequently, 48.1% of the participants had sleep disturbances with 44.9% of them reporting insomnia during the COVID-19 quarantine. Regarding physical activity, there was a decrease in the percentage of participants who exercised from 43.9 % before the pandemic to 25.6% during the pandemic.

**Table 2:** Lifestyle characteristics before and during COVID-19 pandemic.

**Variable**	**Before COVID-19** ***N*** **(%)**	**During COVID-19** ***N*** **(%)**	***p*****-value** **(2-Sided)**
**Physical activity**			
Yes	295 (43.9)^*^	172 (25.6)	0.004
No	377 (56.1)	500 (74.4)	
**Frequency of exercise**			
No exercise	352 (52.4)	453 (67.4)	0.001^*^
1–2/week	147 (21.9)	138 (20.5)	
3–4/week	125 (18.6)	52 (7.7)	
≥5/week	48 (7.1)	29 (4.3)	
**Sleeping hours**			
< 7 h/night	288 (42.9)	449 (66.8)	0.019
7–9 h/night	332 (49.4)	135 (20.1)	
>9 h/night	52 (7.7)	84 (12.5)	
No change		4 (0.6)	
**Smoking**			
Not smoking	506 (75.3)	541 (80.5)	0.000
< 5 cigarettes/day	37 (5.5)	35 (5.2)	
5–10 cigarettes/day	26 (3.9)	103 (15.3)	
>10 cigarettes/day	30 (4.5)	66 (9.8)	
Smoking shisha during quarantine		201 (29.9)	0.000
**Sleep disturbance**			
Yes		323 (48.1)	
No		349 (51.9)	
**Insomnia**			
Yes		302 (44.9)	
No		370 (55.1)	
**SSitting hours during the lockdown:**			
Mean SD		7.3 (4.1)	
Range (Min-Max)		24 (0–24)	

* Data are presented as frequency N (%). Significantly different between at p < 0.05.

About 22.7% of the participants were cigarette smokers before the COVID-19 pandemic, but this percentage decreased to 17.8% during the COVID-19 pandemic. Additionally, a paired *T*-Test accounting for the changes with each participant's change in BMI and weight indicated that participants at a 95% interval consistently increased in BMI and weight while consistently decreasing sleep and exercise during the pandemic when compared to before (Supplementary Figure 1) (*p* ≤ 0.05).

### 3.4. Anthropometric measurements

**Figure 1:**
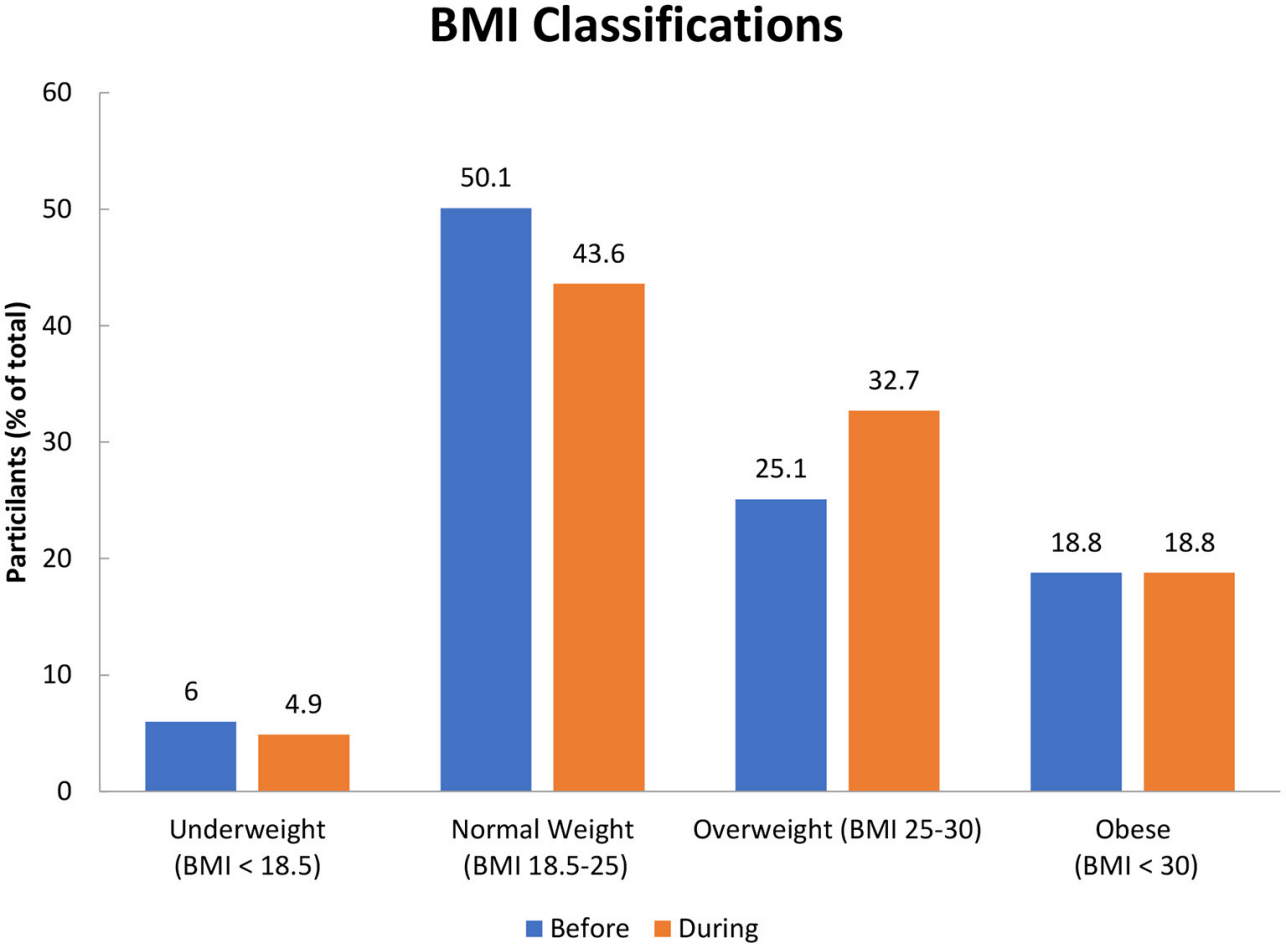
BMI classification before and during COVID-19 outbreaks.

The anthropometric measurement of the participants is represented in Table 3: self-reported weight, height, and the calculated BMI of the participants according to region. As shown, the mean ±SD height for participants in all regions was 167 ± 8.9. Regarding weight, most of the participants had gained weight during the COVID-19 pandemic. The mean ±SD weight (kg) for all participants before the COVID-19 pandemic was 71.2 ± 17.6 and increased to 72.5 ± 17.2 during the COVID-19 pandemic. The mean ± SD BMI (kg/m^2^), for the participants, increased from 25.4 ± 5.8 to 25.9 ± 5.6 during the pandemic. This increased the percentage of people who are overweight (BMI 25.0–29.9 kg/m^2^) by 7.6%, while the obesity prevalence (BMI ≥ 30 kg/m^2^) remained consistent as presented in Figure 1.

**Table 3:** Anthropometric measurement for participants according to the region.

**Variable**		**Total** **(*N* = 672)**	**North** **(*N* = 319)**	**Middle** **(*N* = 223)**	**South** **(*N* = 130)**
**Height (cm)**					
		166.99 ± 8.85	166.13 ± 8.95	166.46 ± 7.76	170.01 ± 9.73^*^
**Weight (kg)**					
	Before COVID-19	71.18 ± 17.55	68.98 ± 17.12	72.23 ± 18.87	74.78 ± 15.52
	During COVID-19	72.92 ± 18.01	69.92 ± 16.78	72.48 ±17.16	78.01 ± 15.19
**BMI (kg/m)** ^2^					
	Before COVID-19	25.4 ± 5.82	24.92 ± 5.57	25.98 ± 6.31	25.93 ± 5.46
	During COVID-19	25.9 ± 5.61	25.27 ± 5.52	26.23 ± 5.90	27.01 ± 5.14

* Data are presented as mean ± standard deviation.

### 3.5. Dietary habits during COVID-19 pandemic

Eating habits during the COVID-19 pandemic are shown in Table 4. A total of 43.5% of the participants self-reported that they changed their eating habits for the worse and 49.1% reported an increased sense of hunger. Almost half (51.5%) of the participants had 3–4 meals/day. We found that 17.7% of participants added snacks to their diet, 14.1% added one meal, and 12.6% reported eating almost all day. Most of the participants (45.7%) reported that they did not meet that recommended water intake by drinking <8 cups/day during the pandemic. Regarding the place of ordering foods, the results revealed that 56.5% of participants ordered groceries from a super or hypermarket.

**Table 4:** Eating habits during COVID-19 pandemic.

**Variable**	**During COVID-19** ***N*** (%)^*^
**Change in lifestyle and eating habits during quarantine**	
Not change	241 (35.9)
Change to better	139 (20.7)
Change to worse	292 (43.5)
**Change in hunger and satiety during COVID-19**	
Not change	240 (35.7)
Increased	330 (49.1)
Decreased	102 (15.2)
**Change number of meals during COVID-19**	
Not change	263 (39.1)
Add one main meal	95 (14.1)
Add snack	119 (17.7)
Skip/remove one or more main meal	83 (12.4)
Skip/remove one or more snacks	27 (4.0)
Almost eating all day	85 (12.6)
**Number of meals /days during COVID-19**	
1–2 meals	198 (29.4)
3–4 meals	346 (51.5)
≥5 meals	128 (19.1)
**Amount of water consumed during COVID-19 quarantine**	
< 1liter/day	307 (45.7)
1–2 liters/day	283 (42.1)
>2 liter/day	82 (12.2)
**Place of ordering foods**	
Not going shopping	134 (19.9)
Minimarket	124 (18.5)
Super or hypermarket	380 (56.5)
Special or organic shop	380 (56.5)
Other	30 (4.5)

* Data are presented as frequency N (%).

The frequency of consumption of food products during the COVID-19 pandemic among adults in Jordan is presented in Table 5. Over half of the participants (51.0%) did not change their consumption of cereals while 32.4% increased their consumption during the pandemic. Additionally, 29.2% increased their protein consumption during the COVID-19 pandemic while 46.4% of the participants had no change in their fruit and vegetable consumption during the pandemic. In our sample, 50.6% of participants reported increasing their consumption of sweets and 22.2% reported increasing their consumption of fat during the pandemic.

**Table 5:** The frequency of consumption of particular foods during the COVID-19 pandemic.

**Food item**	**Increased**	**Decreased**	**No change**	**Not usually eaten before or during COVID-19**
	***N*** **(%)**			
Cereals (rice, spaghetti …)	218 (32.4)	81 (12.1)	343 (51.0)	30 (4.5)
Protein (egg, meat, fish, cheese)	196 (29.2)	47 (7.0)	393 (58.5)	36 (5.4)
Fruit and vegetables	247 (36.8)	85 (12.6)	312 (46.4)	28 (4.2)
Sweet, sugar, and chocolate	340 (50.6)	80 (11.9)	202 (30.1)	50 (7.4)
Fat (butter, oil)	149 (22.2)	58 (8.6)	375 (55.8)	90 (13.4)

The Kruskal-Wallis test showed a statistically significant association in BMI after the pandemic among age, region, education, and marital status (Table 6). The age group of 35 years and older had a significantly (*p* < 0.001) higher BMI than the participants that are younger than 35 years. We found that the Southern region of Jordan had a population with a higher BMI when compared to the Northern and Middle regions of Jordan (*p* < 0.001). Additionally, primary/secondary school-educated individuals had a higher BMI than all other educational attainment levels. Furthermore, the Mann-Whitney *U*-test showed a statistically significant difference in BMI after the pandemic among gender groups. Males were shown to have a higher BMI (26.25 ± 4.89) than females (25.72 ± 6.01) (*p* < 0.05). Moreover, retired individuals had higher BMI (28.60 ± 3.14) with a statistically significant difference (*p* < 0.001).

**Table 6:** Kruskal-Wallis and Mann-Whitney *U*-test of BMI before and during COVID-19 and socio-demographic variables in Jordan.

	**Previous BMI**			**Current BMI**		
	**Mean** ±**SD**	**Median**	***p***	**Mean** ±**SD**	**Median**	***p***
		**(Min-Max)**			**(Min-Max)**	
**Age**						
18–25	23.58 ± 5.50	22.64 (12–48)	< 0.001	23.96 ± 5.61	23.15(14.7–50.9)	< 0.001
25–35	25.72 ± 5.82	24.24 (17–62)		26.18 ± 5.46	24.91 (17–62.4)	
>35	27.71 ± 5.40	27.44 (11–51)		28.26 ± 4.90	28.0 (19.8–52.3)	
**Gender**						
Male	25.69 ± 5.36	24.77 (11–48)	0.088	26.25 ± 4.89	25.93(15.1–42.6)	0.022
Female	25.33 ± 6.09	23.73 (12–62)		25.72 ± 6.01	24.53 (14.7–62.4)	
**Location**						
Rural	25.67 ± 5.66	24.5 (11–54)	0.235	26.32 ± 5.63	26.17 (15.1–56.5)	0.077
Urban	25.37 ± 5.90	24.1 (12–62)		25.72 ± 5.60	24.55 (14.7–62.4)	
**Area**						
North	24.92 ± 5.57	23.53 (16–62)	0.019^*^	25.26 ± 5.52	24.38 (15.1–62.4)	0.001^*^
Middle	25.98 ± 6.30	24.61 (12–51)		26.22 ± 5.90	25.53 (14.7–52.3)	
South	25.93 ± 5.46	24.69 (11–46)		27.01 ± 5.14	27.05 (17.6–45.7)	
**Working**						
Not working	25.96 ± 6.12	24.22 (16–51)	< 0.001	26.09 ± 5.45	25.21 (16.2–52.3)	
Student	23.97 ± 5.57	22.89 (12–48)		24.40 ± 5.69	23.62 (14.7–50.9)	
Work from home	25.82 ± 5.18	24.80 (11–41)		26.85 ± 5.06	26.99 (16.9–42.9)	< 0.001
Work on site	26.25 ± 6.47	24.85 (16–62)		26.63 ± 6.24	25.59 (17.0–62.4)	
Work stopped	25.52 ± 4.00	24.73 (17–39)		26.25 ± 3.37	25.84 (17.5–38.8)	
Retired	29.32 ± 3.14	28.91 (23–35)		28.60 ± 3.14	30.45 (21.3–31.2)	
**Education**						
School	27.92± 6.82	26.04 (14–48)	< 0.001	28.51 ± 5.59	27.77 (20.0–42.6)	< 0.001
University	24.85 ± 5.24	23.82 (11–48)		25.24 ± 5.13	24.49 (14.7–50.9)	
High studies	26.59 ± 6.84	25.39 (12–62)		27.22 ± 6.71	26.44 (16.9–62.4)	
**Marital status**						
Single	24.32 ± 5.44	23.23 (12–51)	< 0.001	24.72 ± 5.44	23.87 (14.7–52.3)	< 0.001
Married	26.77 ± 6.00	25.44 (11–62)		27.29 ± 5.49	26.26 (17.3–62.4)	
Other	26.65 ± 5.35	24.49 (19–38)		27.09 ± 5.91	28.08(20.0–42.9)	

^*^Kruskal-Wallis and Mann-Whitney U test were applied when applicable.

* Statistically significant difference p < 0.05.

**Figure 2:**
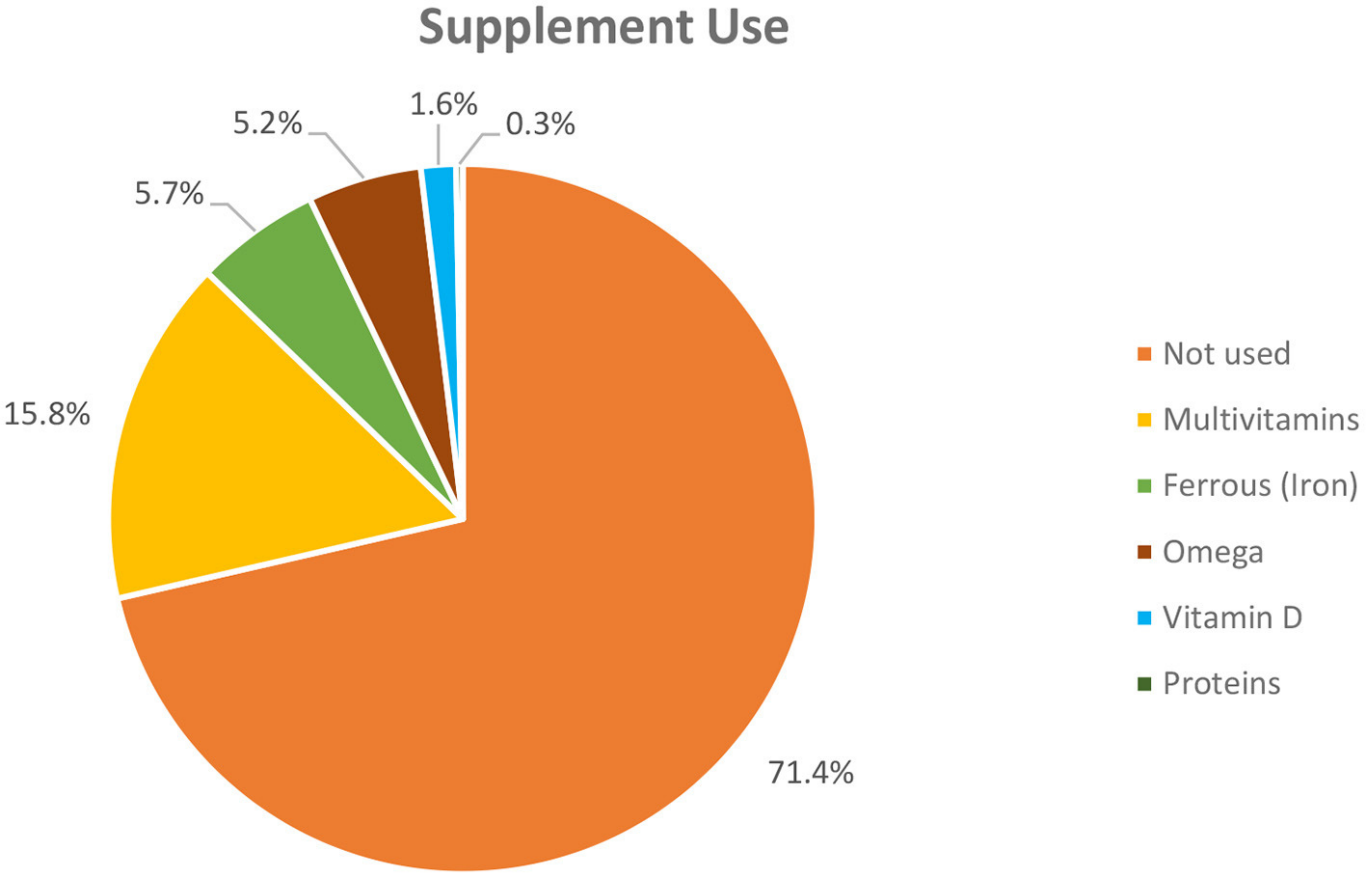
Supplement use among the study participants during the pandemic.

Figure 2 represents the supplement use among study participants as a percentage pie chart. A total of 28.6% of participants reported that they used dietary supplements during the COVID-19 pandemic. Of them, 15.8% used multivitamins supplements, 5.7% used iron supplements, and 5.2% used omega-3 supplements. A total of 1.6% used vitamin D, and only 0.3% used protein supplements. However, the vast majority of participants reported not using any supplements during the pandemic.

### 3.6. Chronic diseases

**Figure 3:**
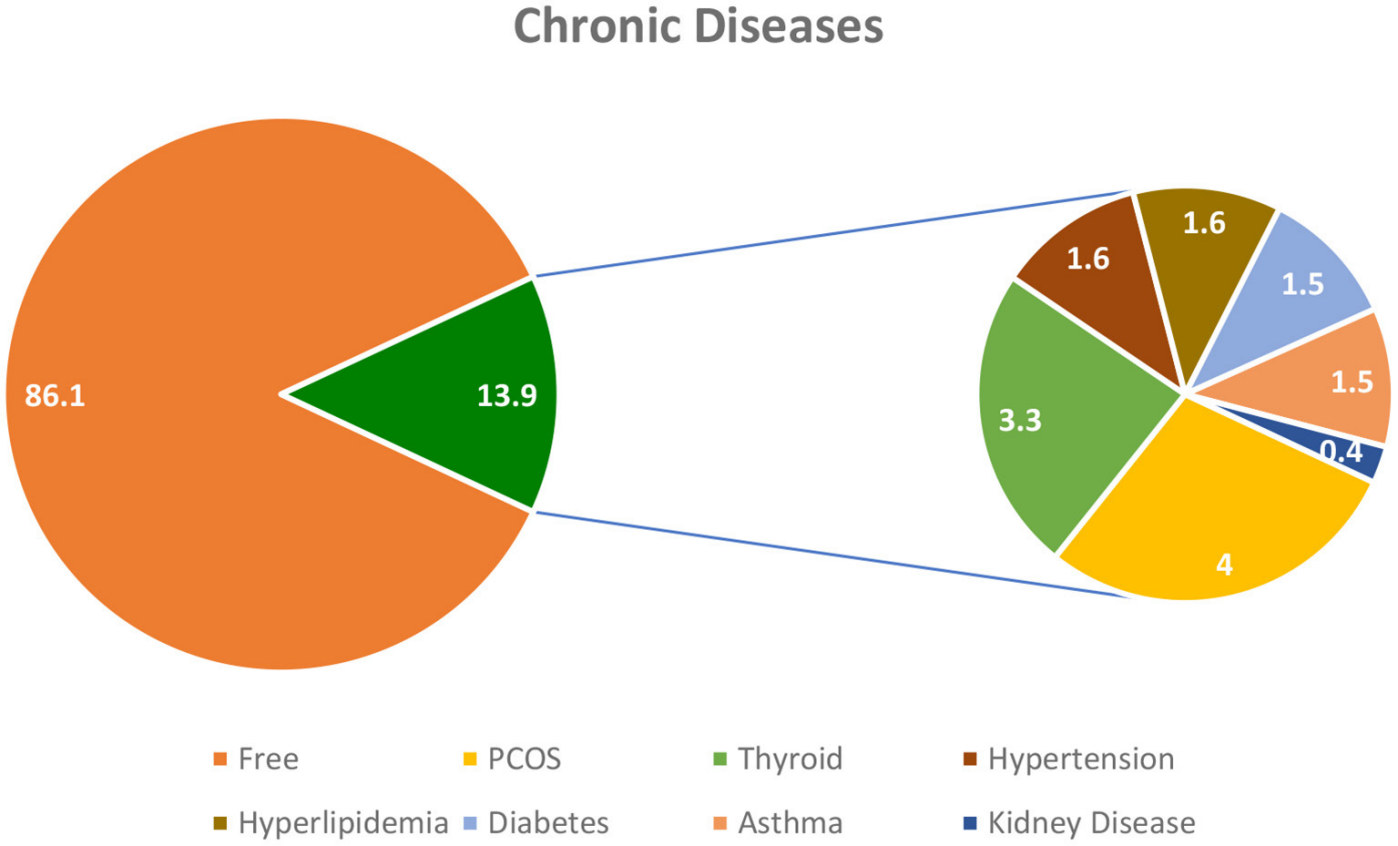
Chronic disease distribution among the study participants.

Figure 3 shows the chronic disease distribution among the participants. The distribution of chronic disease shows that about 86% of the study subjects did not have any other health conditions. Polycystic ovary syndrome (PCOS) was the most prevalent disease among the participants (4%) followed by thyroid disorder (3.3%) and diabetes (1.5%).

## 4. Discussion

The prevalence of obesity has increased significantly with age in both genders. Our results showed that there was a significant difference between participants who were underweight, normal weight, overweight, and obese during the COVID-19 pandemic between age categories (*p* = 0.000). Our results are in agreement with many of the published studies on the trends between age and obesity prevalence. The results of a study performed in the USA from 2015 to 2016 found that the prevalence of obesity was 39.8% among adults and 18.5% among youth (21). They also found that the prevalence of obesity was higher among adults aged 40–59 than among adults aged 20–39 overall and in both men and women (21). Moreover, our results showed that obesity was significantly associated with marital status and education level. We found that married participants were the most likely to be obese (25.8%) followed by divorced or widow participants (23.0%) and then single participants (12.6%) (*p* < 0.001). Similar findings were reported in different studies in Jordan and the USA that found that married people were more likely to be obese than unmarried individuals (*p* < 0.001) (17, 22). The difference in the weight of married participants could partially be attributed to the greater average age of married individuals when compared to the average age of unmarried individuals. Regarding education level, the university-educated participants were more likely to have a normal weight compared to the other education levels. This is comparable to the findings of a recent Jordanian study conducted in 2020 which showed that individuals with adequate health literacy were less likely to have negative health outcomes such as obesity (23). Similar results were obtained from a Spanish cross-sectional study conducted in 2016 which showed that people with a lower level of education had a higher prevalence of obesity associated with poor diet quality (*p* < 0.05) (24).

The Southern region of Jordan had the highest prevalence of overweight (39.2%) and obesity (22.3%). Similar findings were reported in a recent Jordanian study that found that region was significantly associated with obesity (*p* < 0.05) (17). Concerning working status, a significant association with obesity was found in our study (*p* < 0.001). A cross-sectional study conducted in the UAE reported a statistically significant association between working status and lifestyle behavior changes during the COVID-19 pandemic (*p* < 0.05) (20). These observations are also consistent with our findings.

We also observed an overall statistically significant increase in BMI in our sample population as the mean BMI for the participants increased from 25.4 ± 5.82 to 25.9 ± 5.61 during the COVID-19 pandemic (*p* < 0.05) (Table 3). This is comparable with the findings of a recent study which found that BMI significantly increased by 1.51 kg/m^2^ (*p* < 0.001) during the COVID-19 pandemic (25). Another study in Saudi Arabia reported that there was a statistically significant increase in BMI during the pandemic (*p* < 0.05) (26). Moreover, a meta-analysis reported that BMI was statistically significantly higher during the lockdown period (27). On the other hand, a cohort study conducted by Urzeala et al. (28) found that female participants showed normal BMI values during lockdown when compared with males (28).

Regarding lifestyle characteristics, our study reported a significant change in sleeping hours during the pandemic (*p* < 0.019). The number of people who sleep <7 h increased during the pandemic and the number of people who sleep more than 7 h decreased. On the opposite, Di Renzo et al. (29) conducted a study on 3,533 participants and found that the percentage of participants who slept <7 h/night decreased during the pandemic, while the percentage of participants sleeping 7–9 h/night and >9 h/night both increased. Also, 44.9% of the participants reported insomnia (29). A cross-sectional study showed a significant decrease in the percentage of participants who reported sleeping <7 h/day from 51.7% before the pandemic to 39% during the pandemic (*p* < 0.001). Furthermore, 17.3% of participants reported poor sleep quality before the pandemic to 28.1% during the pandemic (*p* < 0.001) (30). In our study, 48.1 % of the participants reported sleep disturbances.

Physical activity was negatively affected during quarantine where most of the participants decreased their exercise levels. Most individuals either did not exercise or reduced their level of physical activity. These results are similar to a cross-sectional study conducted on adult residents of the Middle East and Northern Africa (MENA) region which examined the frequency of physical activity before and during the COVID-19 pandemic (30). This study found that 34.9% of participants did not practice any physical activity before the pandemic, compared to 39.1%during the pandemic (30). Al-Domi et al. (31) found that sedentary behavior was prevalent in Jordan, with 70% (*N* = 2,984) of participants reporting changes in physical activity during the COVID-19 lockdown. And 39% of participants who were obese and overweight reported they were physically inactive (31).

Our study showed that smoking has decreased during the COVID-19 pandemic among participants in Jordan. A study conducted on 3,533 respondents reported a significant difference between smoking habits during the pandemic and found that smoking habits have been reduced during the lockdown (*p* < 0.001) (29). This phenomenon can be explained because of smokers' fear of the increased risk of infection of respiratory distress and mortality from COVID-19 (32).

As shown in Figure 2, 28.6% of participants reported that they used dietary supplements during the COVID-19 pandemic. Abouzid et al. found that the dietary supplements intake for vitamin C, vitamin D, and multivitamins increased significantly during the COVID-19 pandemic. However, they also reported that a high percentage of participants did not use any kind of supplements during the pandemic (33). The Council for Responsible Nutrition reported that Americans increased their consumption of supplements such as multivitamins, vitamin C, and vitamin D, during the COVID-19 pandemic (34). They stated that the main reasons for the increased usage of dietary supplements were to boost the immune system (34). A similar result was found in a cross-sectional study, where the most consumed supplements during the first and second wave of the pandemic were vitamin D (38 and 67%), vitamin C (17 and 37%), and omega-3 fatty acids (15 and 35%) (35).

Most of the study population (43.5%) had self-reported that they changed their eating habits for the worse followed by no change (35.9%) and change for the better (20.7%). These results agree with what was reported by Maffoni et al. that more than one-third of their studied population worsened their lifestyle and eating behavior (36). Most of the subjects (49.1%) reported an increased sense of hunger. Comparable results were found in a cross-sectional study among 4,473 respondents that showed that 44.3% of participants reported an increase in appetite (31). In addition, Di Renzo et al. reported that during the COVID-19 pandemic, the sense of hunger altered for more than half of the population; 17.8% of participants had less appetite, whereas 34.4% of participants experienced an increase in appetite (29).

About 51.5% of the study population had 3–4 meals/day during the COVID-19 pandemic. This result agrees with what was reported by Ismail et al. (30) that the percentage of participants consuming 3–4 meals per day was the highest, followed by 1–2 meals/day, and >5 meals /day (30). These results can likely be related to a lack of appetite, which is common in the presence of depression (30). Most of the participants reported not meeting the recommended water intake by drinking < 2 liters /day during the pandemic. These results are compatible with what was found by Di Renzo et al. who showed that 86.6% of participants reported drinking <2 liters of water per day during the COVID-19 pandemic and 26.2% consumed < 1 liter of water per day (29).

Regarding the place of ordering foods, the results revealed that 56.5% of participants ordered their groceries from super or hypermarkets, followed by 19.9% who reported not going to market, 18.5% reported going to minimarkets, 4.5% using other ways, and 0.6% buying from special or organic shops. These results agree with what was noted by Di Renzo et al. that most of the population purchases food at the supermarket (75.8%) (29). Furthermore, similar results were found in a study conducted in Tunisia where they reported that 64.3% of participants buy their foods from supermarkets, followed by 29.0% who buy their foodstuffs from small shops, and only 2.5% from “souks” which are traditional local markets. This could be explained due to consumers' concern about safety, where supermarkets have implemented several safety measures, such as social distancing, additional cleanliness, and hygiene (37). Over half of the participants (51.0%) did not change their consumption of cereals, 32.4% increased their consumption, 12.1% decreased their cereal consumption, and 4.5% did not eat cereals before or during the pandemic. A comparable result was found in an observational retrospective study in Poland conducted on 312 participants where they found that cereal consumption remains consistent during the COVID-19 pandemic (38). On the other hand, Pellegrini et al. (25) reported that cereal consumption increased during the pandemic (25).

Concerning the result of protein consumption, our study found that 58.5% of participants reported not changing their consumption of protein during the pandemic with 29.2% increasing their consumption. This agrees with what was reported by Bakhsh et al. that 57% of participants admitted no changes in their consumption of protein during the pandemic (meat, fish, and egg) (39). However, Pellegrini et al. reported increased consumption of protein during the pandemic (25). We also found that 46.4% of participants reported not changing their consumption of fruits and vegetables and 36.8% increased their consumption. A similar result was found in a cross-sectional study where they reported that fruit and vegetable consumption remained consistent during the pandemic (40).

We found that 50.6% of the participants increased their consumption of sweets, followed by 30.1% that had no change in their consumption, 11.9% decreased their consumption, and 7.4% did not eat these types of food during the pandemic. Musharbash et al. (41) reported that nearly half of the participants (48.2%) consumed more foods that are rich in sugar (41). In addition, Pellegrini et al. (25) reported an increased sweet consumption during the COVID-19 pandemic (25). Fat consumption results were also comparable with a cross-sectional study conducted on 2,678 respondents, which found that fat consumption remained the same as before the pandemic (42). This could be associated with the increased time spent at home in addition to the sociocultural tendencies of Jordanians in their associations with eating sweets and snacks, especially in this time period. A study conducted in 2022 in Italy investigating the changes in eating habits also indicated that there is a clear trend in the increasing consumption of ultra-processed foods such as sweets (43).

Some of the limitations include the fact that we were not able to independently confirm the data reported both before and after the onset of the pandemic. This was due to the physical and logistical limitations of the lockdown measures at the time of this study and the lack of data in the period immediately before the pandemic. We believe the self-reported data is the best available method of obtaining and interpreting the data for this period. Recall bias and healthy volunteer bias are both sources of error, but we aimed in to limit that as much as possible when formulating the questionnaire to the best of our ability.

## 5. Conclusions

This study highlighted the change in obesity and dietary habits during the COVID-19 pandemic in the Jordanian population. The results can be summarized in the following points:

- COVID-19 pandemic negatively affected the lifestyle of adults in Jordan. Nearly 45% of the population studied had changed eating habits for the worse.
- Most participants reported an increase in weight which corresponded to an overall increase in the prevalence of obesity in the period studied.
- A lack of physical activity was reported in 74.4% of the participants.
- Older age, being married, education level, number of family members, and working status are the variables that are significantly associated with higher BMI in the population.

In conclusion, this data and the results we have obtained underline the need for further action and studies going forward in the region to counteract the trends we have seen. The demographics that displayed the most change in BMI can be more specifically targeted in upcoming efforts to promote more healthy eating and active lifestyles. These results indicate that obesity is continuing to be prevalent in this population and the impact that the pandemic had on that. Future studies can better solidify our knowledge of the prevalence of obesity in Jordan and the Middle East and formulate strategies that best address the factors most contributing to that. These trends are not unique to Jordan and can be observed in various capacities around the world. Understanding the rise in prevalence and the reasons behind it can get us closer to combating these trends and formulating conditions where all people can have healthier weights and lifestyles.

## Data availability statement

The original contributions presented in the study are included in the article/Supplementary material, further inquiries can be directed to the corresponding author.

## Author contributions

All authors listed have made a substantial, direct, and intellectual contribution to the work and approved it for publication.
