# Supplementary figures and images for: Impact of COVID-19 pandemic on obesity among adults in Jordan
Source: Front Nutr. 2023 Jan 20;10:1114076. doi: 10.3389/fnut.2023.1114076 (PMC9896011; doi:10.3389/fnut.2023.1114076)

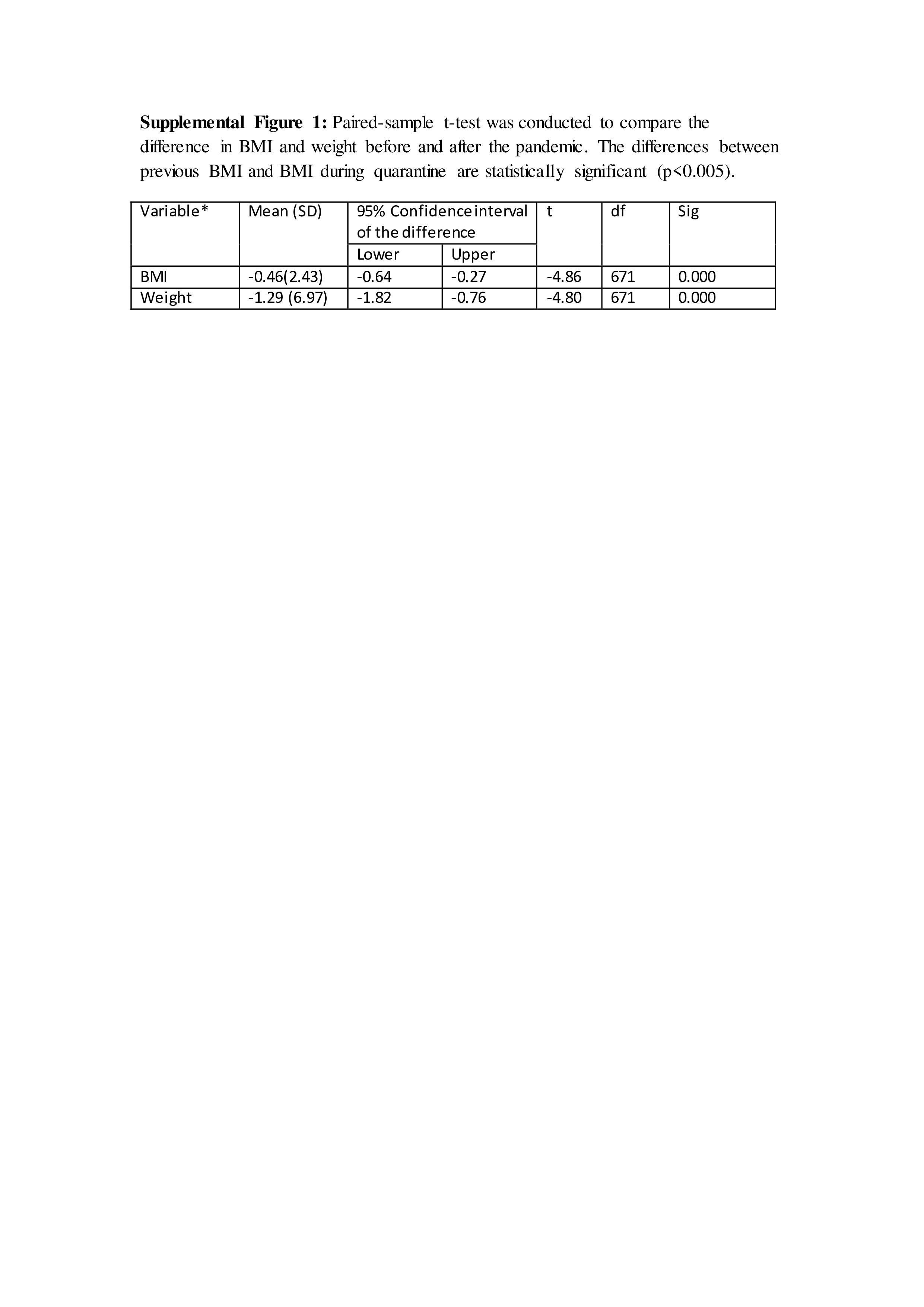

Supplement: Supplementary file 1 [file Image_1.jpg]
